# Impact of 2, 3, 5, 4′-tetrahydroxystilbene-2-O-β-D-glucoside on cognitive deficits in animal models of Alzheimer’s disease: a systematic review

**DOI:** 10.1186/s12906-016-1313-8

**Published:** 2016-08-26

**Authors:** Chenxia Sheng, Weijun Peng, Zeqi Chen, Yucheng Cao, Wei Gong, Zi-an Xia, Yang Wang, Nanxiang Su, Zhe Wang

**Affiliations:** 1 Department of Integrated Traditional Chinese & Western Medicine, The Second Xiangya Hospital, Central South University, No.139 Middle Renmin Road, Changsha, Hunan 410011 People’s Republic of China; 2 Department of Integrated Traditional Chinese & Western Medicine, Xiangya Hospital, Central South University, Changsha, 410008 China

**Keywords:** Alzheimer’s disease, Cognitive deficits, Systematic review, 2, 3, 5, 4′-tetrahydroxystilbene-2-O-β-D-glucoside

## Abstract

**Background:**

The efficacy of 2, 3, 5, 4′-tetrahydroxystilbene-2-O-β-D-glucoside (TSG) treatment on cognitive decline in individuals with Alzheimer’s disease (AD) has not been investigated. Therefore, we systematically reviewed the effect of TSG on cognitive deficits in a rodent model of AD.

**Methods:**

We identified eligible studies published from January 1980 to April 2015 by searching seven electronic databases. We assessed the study quality, evaluated the efficacy of TSG treatment, and performed a stratified meta-analysis and meta-regression analysis to assess the influence of study design on TSG efficacy.

**Results:**

Among a total of 381 publications, 18 fulfilled our inclusion criteria. The overall methodological quality of these studies was poor. The meta-analysis revealed a statistically significant benefit of TSG on acquisition memory (standardized mean difference [SMD] = −1.46 (95 % CI: −1.81 to −1.10, *P* < 0.0001) and retention memory (SMD =1.93 (95 % CI: 1.40 to 2.46, *P* < 0.0001) in experimental models of AD. The stratified analysis revealed a significantly higher effect size for both acquisition and retention memory in studies that used mixed sex models and a significantly higher effect size for acquisition memory in studies that used transgenic models.

**Conclusions:**

Our meta-analysis highlights a significantly better treatment effect in rodent AD models that received TSG that in those that did not. These findings indicate a potential therapeutic role of TSG in AD therapy. However, additional well-designed and detailed experimental studies are needed to evaluate the safety of TSG.

## Background

Alzheimer’s disease (AD) is a major public health problem and a leading cause of disability [1, 2]. The number of people affected by AD is increasing rapidly worldwide, and more than 35 million people currently have AD. By 2050, the prevalence of AD is expected to quadruple to 1 in 85 people, of which 43 % are expected to need a high level of care. World Alzheimer Report 2015 showed that the total estimated worldwide cost of dementia is $818 billion, and it will reach the trillion dollar mark by 2018 [3]. The clinical characteristics of AD are memory loss and impairment of at least one other cognitive domain [4]. Memory dysfunction is generally the first symptom of AD, and it is generally the most severe cognitive impairment. Mounting evidence indicates that the severity of memory dysfunction correlates strongly with the presence of beta-amyloid plaques and intracellular tau and neocortical neurofibrillary tangles [5, 6]. Despite massive research effort to elucidate the causes and mechanisms underlying AD, including recent advances in our understanding of its molecular pathology, effective treatment remains elusive, and none of the existing drugs are able to halt its progression [7, 8]. Consequently, there is a growing interest in new therapeutic strategies for the treatment of AD [9].

Polygonum multiflorum Thunb (PM) is a traditional Chinese herb that has been used widely as an anti-aging drug in the Orient since ancient times. TSG (2, 3, 5, 4′-tetrahydroxystilbene-2-O-β-D-glucoside), a monomer of stilbene, is one of the main components extracted from the root of PM [10]. TSG can cross the blood–brain barrier and has protective effects on hippocampal synaptic plasticity in vitro [11, 12] and in vivo [13]. Recent studies have also shown that TSG reduces the overexpression of amyloid precursor protein (APP) [14], inhibits reactive oxygen species generation [15], and attenuates cognitive impairment in several animal models of AD, including age-advanced rats [11], APP transgenic mice [16], amyloid-β1–42-injected rats [12], and aluminium-exposed rats [14].

No systematic studies have investigated the effect of TSG on cognition in humans with AD. Thus, in the absence of systematic studies investigating TSG in humans, it is not appropriate to state that studies are needed to confirm the benefits of TSG because there are no findings to confirm. It is more appropriate to state that studies must be conducted to identify a potential benefit. Systematic reviews of animal studies synthesize the available evidence in an unbiased manner to provide evidence for the potential translational value of effective therapeutic interventions in animal models to humans [17], contribute to models of clinically relevant problems, and facilitate decisions regarding the design and conduct of subsequent human clinical trials [18]. Therefore, the aim of the current study was to perform a robust systematic review and meta-analysis of all available experimental evidence concerning the effects of TSG on cognitive impairment in animal models of AD and to provide an evidence-based foundation for future clinical trials.

## Methods

### Literature search

On April 3, 2015 we searched seven electronic databases (PubMed, Web of Science, MEDLINE, Google Scholar, Embase, CNKI, and Wanfang data). All searches were restricted to literature published between January 1980 and April 2015. The following terms were included in the searches: “Alzheimer’s disease” (or “Alzheimer disease”, “dementia”, “Alzheimer”, “Alzheimers” or “Alzheimer’s”) and “tetrahydroxystilbene glucoside” (or “Polygonum multiflorum Thunb”, “Radix Polygonum Multiform”, “tetrahydroxy stilbene glucoside”, “2, 3, 5, 4′-tetrahydroxystilbene-2-O-β-D-glucoside”, or “TSG”). We limited our search results to animal studies. Additional relevant publications were identified from the reference lists of the resulting research articles and reviews. Bias was prevented by the a priori defined inclusion and exclusion criteria described in Table 1. Two investigators (SC and PW) assessed the titles and abstracts of the studies and obtained copies of the articles describing controlled studies of TSG or its analogues in animal models of AD.

**Table 1:** Criteria for study inclusion/exclusion

Inclusion criteria	Exclusion criteria
(1) TSG were administered.	(1) TSG were not administered.
(2) Experimental AD was induced in rodents.	(2) Other types of animals (e.g., sheep, cats, and dogs) were used.
(3) treatment group was treated with TSG, and control group was administered a placebo.	(3) Treatment group was administered another neuroprotective agent in addition to TSG.
(4) Cognitive function was measured by the MWM, passageway water maze, passive avoidance task, Y maze experiment etc.	(4) Treatment group was administered another Chinese Traditional Medicine in addition to TSG.
(5) Article was published in English or Chinese language.	(5) Only biochemical or physiological outcomes of treatment efficacy were assessed.
	(6) No control group was used.
	(7) Duplicate publications or data presented in duplicate by additional publications.

### Data extraction

The following information was extracted from each included study by two investigators: animal species; sex; type of AD model; sample size; dose, method, and timing of TSG administration; main experimental groups; intervention regime (i.e., administration route and number of injections); and cognitive outcome assessments.

Any studies that reported effects of TSG on learning and memory abilities using an animal model of AD were included. The cognitive outcomes were assessed by the Morris water maze, passageway water maze, passive avoidance task, and Y maze experiment, among others, which are commonly used to evaluate spatial learning/memory in both mice and rats [19, 20]. The details of the individual study characteristics were extracted from each publication. When a single publication included groups with different TSG doses or different AD models across groups, these data were extracted and considered independent experiments. Because the learning trials to assess memory function were conducted over 5 days, the final test indicates the learning ability of rats/mice [21]. Therefore, we extracted the data for the final time point only when memory function was assessed at a different time point. If any information was missing, then the study investigators attempted to obtain the information from the study authors. If these data were not available, then we excluded the study from the analysis. If the data were presented in graphical form only, then we contacted the authors to request the numerical values. If numerical values could not be obtained, then the numerical values were estimated from the graphs using digital ruler software.

### Methodological study quality

The methodological quality of the studies was assessed based on a checklist of the Collaborative Approach to Meta-Analysis and Review of Animal Data from Experimental Studies (CAMARADES), as previously described, with minor modifications [22]. One point was assigned for written evidence of each of the criteria described in Table 2.

**Table 2:** The CAMARADES quality items

Authors & Year	➀	➁	➂	➃	➄	➅	➆	➇	➈	➉	Quality score
Zhang et al. 2006 [17]	√	√	√	√			√	√		√	7
Zhou et al. 2012 [13]	√	√			√		√	√	√	√	7
Zhang et al. 2006 [27]	√	√	√				√	√		√	6
Xing et al. 2006 [28]	√	√	√				√	√		√	6
Xie et al. 2005 [29]	√						√	√		√	4
Chu et al. 2005 [30]	√	√	√				√	√		√	6
Huang et al. 2008 [31]	√	√	√					√		√	5
Huang et al. 2008 [32]	√	√	√					√		√	5
Liu et al. 2012 [33]	√	√	√				√	√		√	6
Chu et al. 2004 [34]	√	√					√	√		√	5
Ye et al. 2003 [35]	√		√				√	√		√	5
Ye et al. 2005 [36]	√	√	√				√	√		√	6
Wang et al. 2007 [12]	√	√					√	√		√	6
Luo et al. 2009 [15]	√	√			√		√	√	√	√	7
Hou et al. 2011 [40]	√	√			√		√	√	√	√	7
Luo et al. 2010 [37]	√	√	√					√		√	5
Zhao et al. 2004 [38]	√	√			√		√	√	√	√	7
Luo et al. 2012 [39]	√	√					√	√		√	5

(1) peer reviewed publication; (2) presence of randomization of subjects into treatment groups; (3) assessment of dose–response relationship; (4) blinded assessment of behavioral outcome; (5) monitoring of physiological parameters such as body temperature; (6) calculation of necessary sample size to achieve sufficient power; (7) statement of compliance with animal welfare regulations; (8) avoidance of anesthetic agents with marked intrinsic neuroprotective properties (e.g., ketamine); (9) statement of potential conflict of interests; (10) use of a suitable animal model

Although a large number of tools is currently used to assess the quality of animal studies, most instruments assess study quality and internal and external validity simultaneously [23]. No tools that have been identified that are able to assess internal validity alone. Therefore, in addition to the modified CAMARADES checklist, we used another previously described checklist [24, 25] to assess study quality based on the study characteristics, such as the age, species, and sex of the animals used, and the dose and duration of TSG supplementation (Table 3). The quality of all studies was assessed independently by two reviewers (PW and SC).

**Table 3:** The second quality items

Study Quality:	Zhang et al. 2006 [17]	Zhou et al. 2012 [13]	Zhang et al. 2006 [27]	Xing et al. 2006 [28]	Xie et al. 2005 [29]	Chu et al. 2005 [30]	Huang et al. 2008 [31]	Huang et al. 2008 [32]	Liu et al. 2012 [33]	Chu et al. 2004 [34]	Ye et al. 2003 [35]	Ye et al. 2005 [36]	Wang et al. 2007 [12]	Luo et al. 2009 [15]	Hou et al. 2011 [40]	Luo et al. 2010 [37]	Zhao et al. 2004 [38]	Luo et al. 2012 [39]
Research question specified and clear?	√	√	√	√	√	√	√	√	√	√	√	√	√	√	√	√	√	√
Outcome measures relevant for AD research	√	√	√	√	√	√	√	√	√	√	√	√	√	√	√	√	√	√
Are the characteristics of study population clear?																		
Species	√	√	√	√	√	√	√	√	√	√	√	√	√	√	√	√	√	√
Background/generation	√	√	√	√	√	√	√	√	√	√	√	√	√	√	√	√	√	√
Sex (and distribution)	√	√	√	√	√	N	√	N	√	√	√	√	√	√	√	√	√	√
Age	√	N	√	√	√	√	N	√	N	√	√	√	√	N	√	N	N	√
Presence and correct control group?	√	√	√	√	√	√	√	√	√	√	√	√	√	√	√	√	√	√
Where the groups similar at baseline (if not randomized think of weight and sex etc.)?	√	√	√	√	N	√	√	√	√	√	N	√	?	√	√	√	√	√
Is the experiment randomized?	√	√	√	√	N	√	√	√	√	√	N	√	√	√	√	√	√	√
Kind of supplement mentioned (TSG)?	√	√	√	√	√	√	√	√	√	√	√	√	√	√	√	√	√	√
Age when supplementation started mentioned?	√	N	√	√	√	√	N	√	N	√	√	√	√	N	√	N	N	√
Duration of supplementation clear and specified?	√	√	√	√	√	√	√	√	√	√	√	√	√	√	√	√	√	√
Amount of TSG mentioned	√	√	√	√	√	√	√	√	√	√	√	√	√	√	√	√	√	√
Administration route specified	√	√	√	√	√	√	√	√	√	√	√	√	√	√	√	√	√	√
Is the timing of the supplementation during the day specified and similar in both groups?	√	√	√	√	√	√	√	√	√	√	√	√	√	√	√	√	√	√
Methods used for outcome assessment the same in both groups ?	√	√	√	√	√	√	√	√	√	√	√	√	√	√	√	√	√	√
Drop outs described for each group separately?	N	N	N	N	N	N	N	N	N	√	N	N	N	N	N	N	N	N
Blinded outcome assessment?	√	N	N	N	N	N	N	N	N	N	N	N	N	N	N	N	N	N
Was the outcome assessment randomized across the groups?	N	N	N	N	N	N	N	N	N	N	N	N	N	N	N	N	N	N
Total number of animals included in statistical analyses clear?	√	√	√	√	√	√	√	√	√	√	√	√	√	√	√	√	√	√
Age of sacrificing animals mentioned?	√	N	√	√	√	√	N	√	N	√	N	√	√	N	√	N	N	√
Quality score (items√)	19	15	18	18	16	17	16	17	16	19	15	18	18	15	15	15	15	18

### Statistical analysis

The global estimated effect of TSG treatment on cognitive outcomes was calculated using the standardized mean difference (SMD) and 95 % confidence intervals (CI), which is used as a summary statistic in meta-analyses when studies assess the same outcome but measure the outcome in different ways, based on the guidelines in the Cochrane Handbook for Systematic Reviews of Interventions [26]. The SMD is equal to the difference in mean outcomes between groups divided by the standard deviation of outcomes among participants and is reported in units of standard deviation. Negative SMD effect sizes indicate a positive efficacy for acquisition memory, whereas positive SMD effect sizes indicate a positive efficacy for retention memory. Within- and between-study heterogeneity was evaluated using Cochran’s Q-statistic, *P* < 0.10, to indicate heterogeneity among studies [25]. The statistical heterogeneity across studies was assessed using the *I*^2^ statistic, with values of 75, 50, and 25 % representing high, moderate, and low heterogeneity, respectively. A value ≥50 % suggests unacceptable heterogeneity among the studies [27]. A random-effects model was used to pool the SMD when the heterogeneity was significant (*I*^2^ ≥ 50 %); otherwise, a fixed effects model was applied.

Subgroup analyses were also used to identify associations between relevant study characteristics, such as species, sex, TSG dose, and study quality, as possible sources of heterogeneity. Heterogeneity and the *x*^2^ distribution with n-1° of freedom (df), where n equals the number of groups, was used to assess the differences in mean effect sizes between groups. To adjust the values for multiple comparisons, we used Bonferroni’s correction methods (declared significance =1 − (1 − denoted significance)∧(1/number of comparisons)), which was appropriate for the number of analyses conducted [28]. The denoted significance level was set at *P* < 0.05. The declared *P* values for this study were 0.0017 for acquisition memory and 0.0037 for retention memory.

Finally, meta-regression analyses were conducted to reveal potential sources of heterogeneity in the efficacy of TSG when high heterogeneity was present. The following variables were included in the meta-regression analyses: species, sex, TSG dose, and study quality. To allow for multiple comparisons, the significance was set at *P* < 0.01.

All statistical analyses were performed using the Stata software package (version 13.0) and Review Manager (version 5.3).

## Results

### Study inclusion

**Fig. 1:**
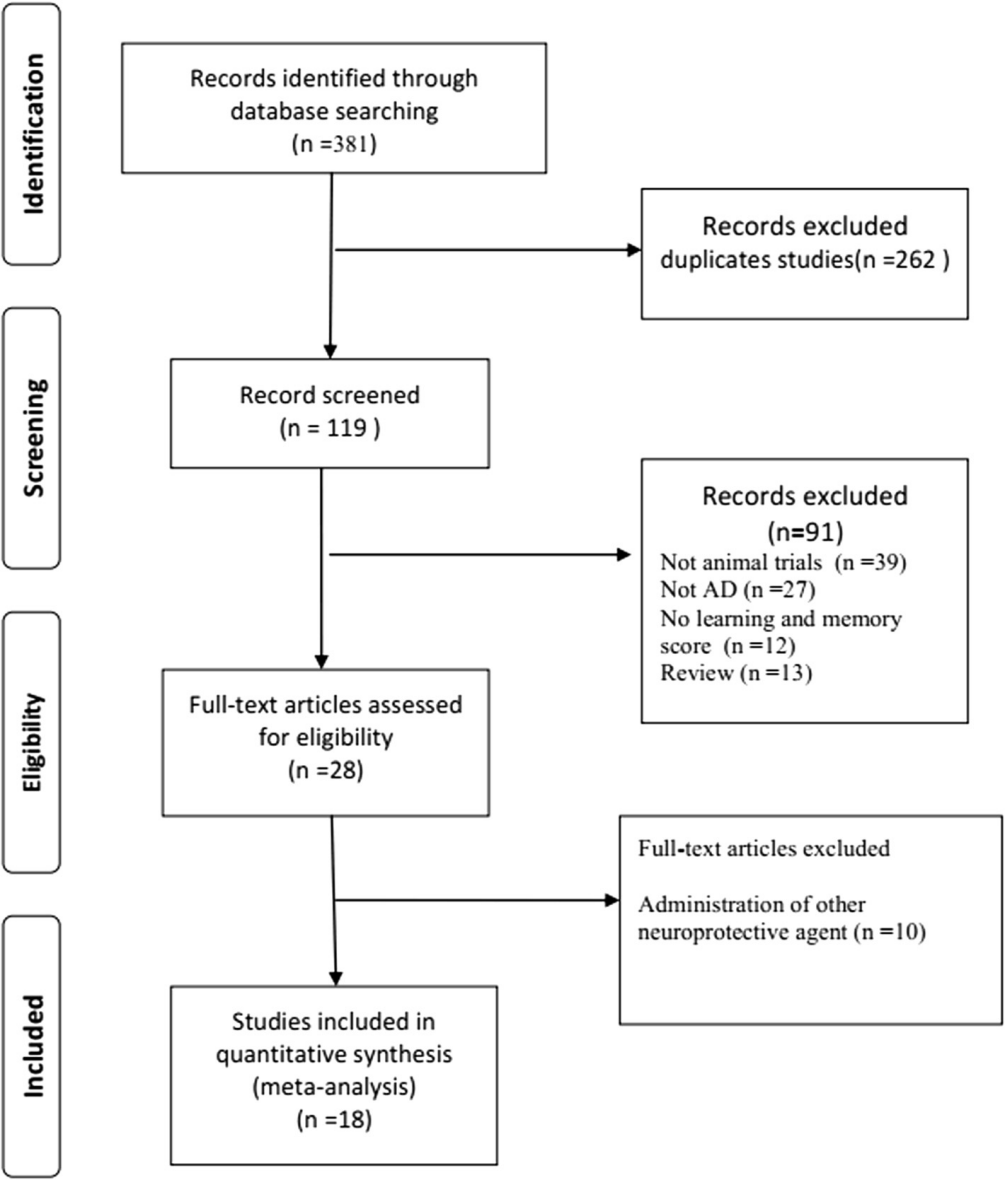
Flow diagram of the study search process

A total of 381 publications were identified, of which 18 met our inclusion criteria [11, 12, 14, 16, 29–42]. Our meta-analysis is based on these 18 studies, which include 39 comparisons of acquisition memory and 15 comparisons of retention memory (Fig. 1).

### Study characteristics

Of the 18 included studies (Table 4), 13 were published in Chinese academic journals and the remainder were published in English. The characteristics of these studies are presented in Table 1. A total of 10 studies used mice (3 Balb/c mice [31, 32, 36], 2 Kunming mice [33, 38], 3 PDAPPV717I transgenic mice [16, 29, 30], and 2 senescence accelerated prone mice/8 [34, 35]), 7 studies used Sprague-Dawley rats [11, 12, 14, 37, 39, 41, 42] and 1 study used Wistar rats [40]. Female animals were used in 3 studies [31, 32, 36], male animals were used in 11 studies [11, 12, 14, 34, 35, 37–42], and both males and females were used in 4 studies [16, 29, 30, 33]. Five studies used a transgenic model [16, 29, 30, 34, 35], 3 studies used a D-galactose-induced model [31–33], 2 studies used a cholinergic damage model [37, 38], 2 studies used an age-advanced model [11, 42], 5 studies used an amyloid-β1-42-injected model [12, 36, 39, 41, 42], 1 study used an aluminium chloride-exposed model [14], and 1 study used a hypercholesterolemia model [40]. To assess learning and memory, 14 studies used the Morris water maze test, and all of these studies used a hidden platform during the probe phase [12, 16, 29–38, 40, 42]. One study used a passageway water maze [11], 1 study used passive avoidance task [14], and 2 studies used a Y maze experiment [39, 41].

**Table 4:** Characteristics of included studies

Authors & Year	Species & sex (no.)	Sex	Type of mode	Drug (treated/control)	Main experimental groups	Dose of administration	Method/Time of TSG administration	Quality score	Outcome
Zhang et al. 2006 [17]	PDAPPV717I transgenic mouse (72)	Female & Male	Transgenic model	TSG/water	1) AD plus water	120 (TSG -L), & 240 (TSG-H) mg/kg body wt	Intragastrically/4 months	7	MWM
					2) AD plus TSG -L				
					3) AD plus TSG-H				
Zhou et al. 2012 [13]	Sprague-Dawley rat (*n* = 12)	Male	Aβ infused rats	TSG/NS	1) AD plus NS	25 mg/kg body wt	Intragastrically/4 months	7	MWM
					2) AD plus TSG				
Zhang et al. 2006 [27]	PDAPPV717I transgenic mouse (53)	Female & Male	Transgenic model	TSG/water	1) AD plus water	50 mg (TSG -L), 100 mg (TSG -M),& 200 mg (TSG-H) g/kg body wt	Intragastrically/4 months	6	MWM
					2) AD plus TSG -L				
					3) AD plus TSG -M				
					4) AD plus TSG-H				
Xing et al. 2006 [28]	PDAPPV717I transgenic mouse (46)	Female & Male	Transgenic model	TSG/water	1) AD plus water	0.05 (TSG -L), & 0.20 (TSG-H) g/kg body wt	Intragastrically/6 months	6	MWM
					2) AD plus TSG -L				
					3) AD plus TSG-H				
Xie et al. 2005 [29]	BALB/c mouse (*n* = 12)	Female	D-galactose infused mice	TSG/water	1) AD plus water	0.05 g/kg body wt	Intragastrically/2 months	4	MWM
					2) AD plus TSG				
Chu et al. 2005 [30]	Balb/c mouse (*n* = 52)	Female	D-galactose infused mice	TSG/water	1) AD plus water	33 mg (TSG-L), 100 mg (TSG-M),& 300 mg (TSG-H) g/kg body wt	Intragastrically/2 months	6	MWM
					2) AD plus TSG -L				
					3) AD plus TSG -M				
					4) AD plus TSG-H				
Huang et al. 2008 [31]	Kunming mouse (*n* = 40)	Female & Male	D-galactose Infused mice	TSG/water	1) AD plus water	33 mg (TSG-L), 100 mg (TSG-M),& 300 mg (TSG-H) g/kg body wt	Intragastrically/2 months	5	MWM
					2) AD plus TSG –L				
					3) AD plus TSG –M				
					4) AD plus TSG-H				
Huang et al. 2008 [32]	SMAP mouse	Male	Transgenic model	TSG/NS	1) AD plus NS	33 mg (TSG-L), 100 mg (TSG-M),& 300 mg (TSG-H) g/kg body wt	Intragastrically/50 days	5	MWM
					2) AD plus TSG –L				
					3) AD plus TSG –M				
					4) AD plus				
					TSG-H				
Liu et al. 2012 [33]	SMAP mouse	Male	Transgenic model	TSG/NS	1) AD plus NS	33 mg (TSG-L), 100 mg (TSG-M),& 300 mg (TSG-H) g/kg body wt	Intragastrically/50 days	6	MWM
					2) AD plus TSG –L				
					3) AD plus TSG –M				
					4) AD plus TSG-H				
Chu et al. 2004 [34]	Balb/c mouse (*n* = 52)	Female	Aβ infused mice	TSG/NS	1) AD plus NS	0.1 g/kg body wt	Intragastrically/8 weeks	5	MWM
					2) AD plus TSG				
Ye et al. 2003 [35]	Sprague-Dawley rat (*n* = 43)	Male	*ibotenic acid* infused rats	TSG/NS	1) AD plus water	30 mg (TSG-L), 60 mg (TSG-M),& 120 mg (TSG-H) g/kg body wt	Intraperitoneally/1 month	5	MWM
					2) AD plus TSG -L				
					3) AD plus TSG -M				
					4) AD plus TSG-H				
Ye et al. 2005 [36]	Sprague-Dawley rat (*n* = 29)	Male	scopolamineinfused rats	TSG/NS	1) AD plus water	33 mg (TSG-L) & 100 mg (TSG-H) g/kg body wt	Intraperitoneally/2 months	6	MWM
					2) AD plus TSG -L				
					3) AD plus TSG-H				
Wang et al. 2007 [12]	Sprague-Dawley rat	Male	Aged rats	TSG/water	1) AD plus water	30 mg(TSG-L), 60 mg (TSG-M) g/kg body wt	Intragastrically/12 W	6	PWM
					2) AD plus TSG -L				
					3) AD plus TSG-H				
Luo et al. 2009 [15]	Sprague-Dawley rat	Male	aluminum chloride exposure rats	TSG/water	1) AD plus water	4000 mg g/kg body wt	Intragastrically/20 W	7	PAT
					2) AD plus TSG				
Hou et al. 2011 [40]	Sprague-Dawley rat	Male	Aged rats	TSG/NS	1) AD plus NS	50 mg g/kg body wt	Intragastrically/20 W	7	MWM
					2) AD plus TSG				
Luo et al. 2010 [37]	Sprague-Dawley rat	Male	Aβ infused rats	TSG/NS	1) AD plus NS	100 mg g/kg body wt	Intragastrically/3 W	5	YEM
					2) AD plus TSG				
Zhao et al. 2004 [38]	Wistar rat	Male	Hypercholestero-lemia rats	TSG/NS	1) AD plus NS	30 mg (TSG-L), 60 mg (TSG-M),& 120 mg (TSG-H) g/kg body wt	Intragastrically/10 W	7	MWM
					2) AD plus TSG -L				
					3) AD plus TSG -M				
					4) AD plus TSG-H				
Luo et al. 2012 [39]	Sprague-Dawley rat	Male	Aβ infused rats	TSG/NS	1) AD plus NS	50 mg g/kg body wt	Intragastrically/3 W	4	YEM
					2) AD plus TSG				

*MWM* Morris water maze test, *PWM* Passageway water maze, *PAT* Passive avoidance task, *YEM* Y maze experiment

### Study quality

According to the modified CAMARADES checklist, the median quality score for the 18 included studies was poor (5.692; interquartile range: 5–6), with scores ranging from 4 to 7. No study received a score of 0 or 10. Five studies received scores indicating high quality [12, 14, 16, 39, 42]. One study reported monitoring of physiological parameters [12]. One study mentioned allocation concealment [16]. Two studies [31, 37] did not report randomization of animals into treatment groups. Ten studies [16, 29, 30, 32–35, 37, 38, 40] assessed dose-response relationships. Four studies [12, 14, 39, 42] stated no potential conflicts of interest. Unfortunately, no studies described the calculation of the sample size required to achieve sufficient power to detect differences.

According to our secondary criteria, the average quality score of the included studies was 16.74, with scores ranging from 15 to 19. Six studies [12, 14, 37, 39, 40, 42] received a score of 15, and two studies received a score of 19 [16, 36]. Six studies did not report the age of the animals [12, 14, 37, 39, 40, 42]. Only one study [16] reported blinded outcome assessments. No studies mentioned any dropouts. No studies mentioned whether the order of the outcome assessments was randomized across groups.

### Overall efficacy

**Fig. 2:**
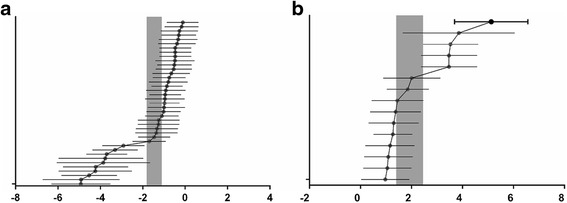
Effects of TSG on acquisition memory (**a**) and retention memory (**b**). The horizontal lines represent the mean estimated effect sizes and 95 % CIs for each comparison. The vertical grey bars represent the 95 % CIs of the pooled estimated effect sizes

For acquisition memory, the global estimated effect of TSG was −1.46 (95 % CI: −1.81 to −1.10, *P* < 0.0001), with significant heterogeneity among studies (heterogeneity: *x*^2^ = 216.17, df = 38, *P* < 0.00001, *I*^2^ = 82 %; Fig. 2a). For retention memory, the global estimated effect of TSG was 1.93 (95 % CI: 1.40 to 2.46, *P* < 0.0001), with significant heterogeneity among studies (*x*^2^ = 56.97, df = 14, *P* < 0.0001; *I*^2^ = 75 %; Fig. 2b).

### Stratified meta-analysis

Subgroup analyses were conducted to assess the degree to which the methodological differences between trials may have systematically influenced differences observed in the primary treatment outcomes. The results of the stratified analyses are described in Table 5.

**Table 5:** The results of stratified meta-analysis

Subgroups	Acquisition memory				Retention memory			
	Studies	Participants	Effect size [95 % CI]	Subgroup differences	Dtudies	Participants	Effect size [95 % CI]	Subgroup differences
Animal species								
APP mice	11	349	−1.84 [−2.62, −1.05]	*P* = 0.0002	4	144	2.84 [1.35, 4.32]	*P* < 0.00001
Balb/c mice	5	124	−0.45 [−0.80, −0.09]					
Kunming mice	5	98	−0.84 [−1.27, −0.42]		3	60	1.12 [0.56, 1.67]	
SMP8 mice	6	120	−0.79 [−1.17, −0.41]		6	120	1.36 [0.95, 1.77]	
SD rats	9	208	−2.78 [−4.06, −1.51]		2	48	3.60 [2.63, 4.57]	
Wistar rats	3	105	−1.35 [−1.17, −0.41]					
Sex								
Male	20	471	−1.59 [−2.12, −1.06]	*P* < 0.00001	8	168	1.61 [1.17, 2.45]	*P* = 0.65
Female	5	124	−0.45 [−0.80, −0.09]					
Female & Male	14	409	−1.68 [−2.31, −1.05]		7	204	2.06 [1.15, 2.98]	
Model								
Untransgenic	22	535	−1.46 [−1.94, −0.98]	*P* = 0.99	5	108	1.98 [0.88,3.08]	*P* = 0.93
Transgenic	17	469	−1.46 [−2.00, −0.91]		10	264	1.92 [1.28, 2.56]	
Dose								
Less 100 mg	21	582	−1.92 [−2.52, −1.32]	*P* = 0.04	8	216	2.23 [1.34, 3.11]	*P* = 0.15
100 mg	11	270	−0.91 [−1.37, −0.45]		3	60	1.25 [0.69, 1.82]	
200 mg	2	64	0.92 [−1.46, −0.39]					
300 mg	4	88	−1.01 [−1.46, −0.56]		4	96	1.98 [0.92, 3.05]	
Quality								
4	2	60	−2.10 [−5.27, −1.08]	*P* < 0.00001	1	12	3.86 [1.66, 6.05]	*P* = 0.006
5	15	361	−1.26 [−1.64, −0.81]		6	120	1.15 [0.76, 1.55]	
6	15	387	−0.63 [−0.83, −0.42]		3	60	1.56 [0.96, 2.16]	
7	7	196	−3.82 [−4.41, −3.23]		5	180	2.97 [1.71, 4.22]

**Table 6:** Metaregression analysis to identify sources of bias associated with study characteristics

Covariates	Coef.	Std. err.	t	*P* > |t|	[95 % conf. interval]	
(a) Acquisition memory						
Quality score	−.8748308	.2568104	−3.41	0.002	−1.400067	−.3495946
sex	.6720335	.4156478	1.62	0.117	−.1780618	1.522129
dose	.1827889	. 2101048	0.87	0.391	−.2469237	.6125015
special	.24532	.1328595	1.85	0.075	−.0264082	.5170483
model	.8326842	.7477604	1.11	0.294	−.8588674	2.524236
(b) Retention memory						
Quality score	.4047783	.3156139	1.28	0.224	−.2828853	1.092442
sex	.7864006	.291455	2.70	0.019	1.421426	.1513747
dose	−.4543919	.3441781	−1.32	0.211	−1.204292	.2955078
special	.7864006	.291455	2.70	0.019	.1513747	1.421426

**Fig. 3:**
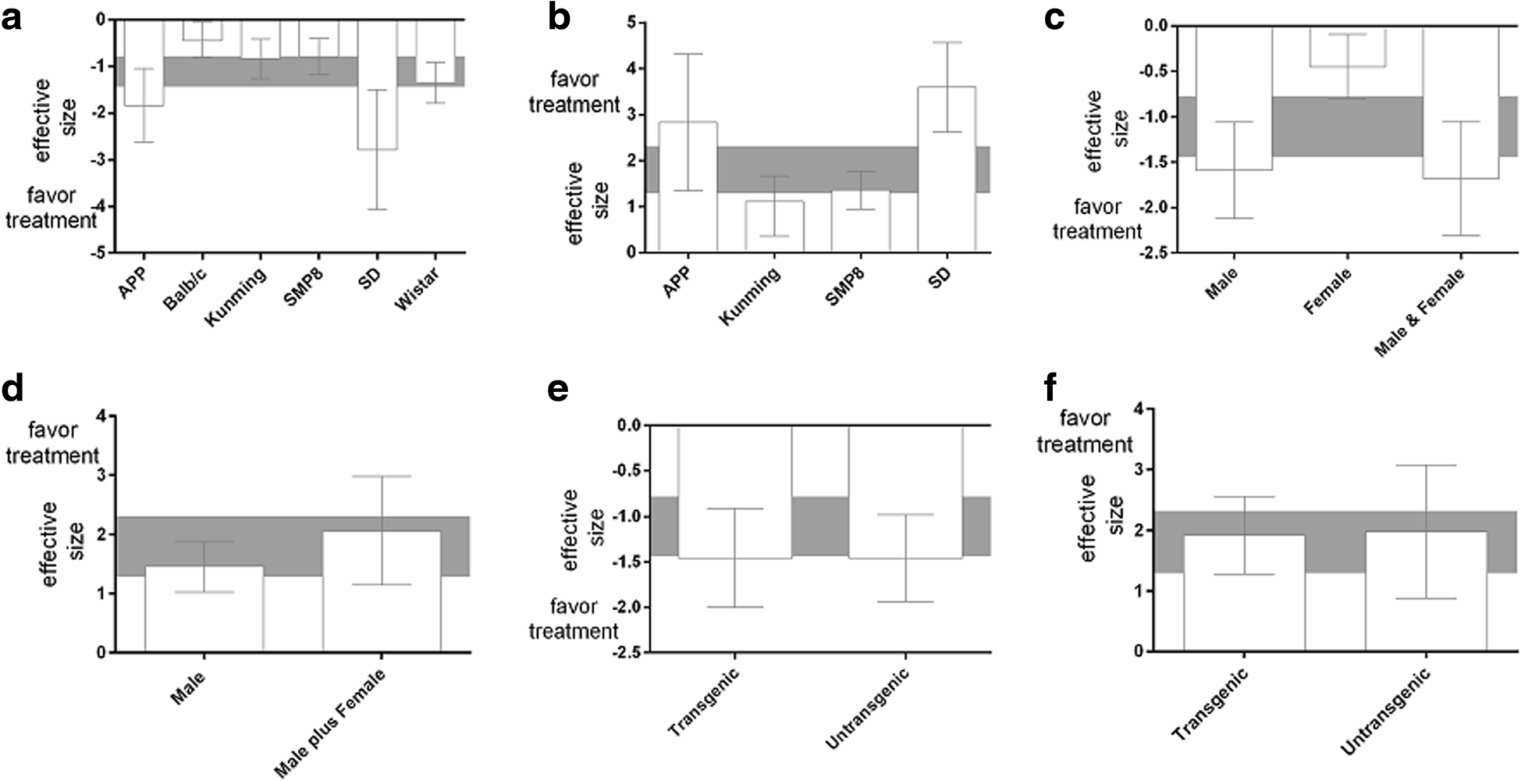
Effect size stratified by animal species for (**a**) acquisition memory and (**b**) retention memory, according to animal gender. Effect size stratified by gender for (**c**) acquisition memory and (**d**) retention memory stratified by the model method for (**e**) acquisition memory and (**f**) retention memory. Grey bands represent the 95 % CIs for the global estimated effect sizes

We examined the protective effects of TSG on different rodent species. For both acquisition and retention memory, the effect size was significantly higher in studies that used Sprague-Dawley rats than in studies that used other species (Fig. 3a and b); *P* = 0.0002 for acquisition memory and *P* < 0.00001 for retention memory, respectively). The effect size was −2.78 (95 % CI: −4.06 to −1.51) for acquisition memory and 3.60 (95 % CI: 2.63 to 4.57) for retention memory in studies that used Sprague-Dawley rats.

We also examined the effect size of TSG on acquisition memory and retention memory in studies that used male, female, or mixed sex animals. The effect size on acquisition memory was significantly higher in studies that used mixed sex animals than in those that used male or female animals only (*x*^2^ = 18.45, df = 2, *P* < 0.00001; Fig. 3c). The effect size on retention memory was examined in studies that used mixed sex or male animals only because limited data were available from studies with female animals only. The effect size was higher in studies that used mixed sex animals (−2.06, 95 % CI: 1.15 to 2.98) than in those that used male animals only, but this difference was not significant (*x*^2^ = 0.21, df = 1, *P* = 0.65; Fig. 3d). A significant effect size for acquisition memory was observed in both transgenic models (−1.46, 95 % CI: −1.94 to −0.98, *P* < 0.0001) and non-transgenic models (−1.46, 95 % CI: −2.00 to −0.91, *P* < 0.0001); no significant difference was observed between models (*x*^2^ = 0.00, df = 1, *P* = 0.99; Fig. 3e). A slightly higher effect size for retention memory was observed in non-transgenic models than in transgenic models, but no significant differences were observed between models (*x*^2^ = 0.001, df = 1, *P* = 0.93; Fig. 3f).

**Fig. 4:**
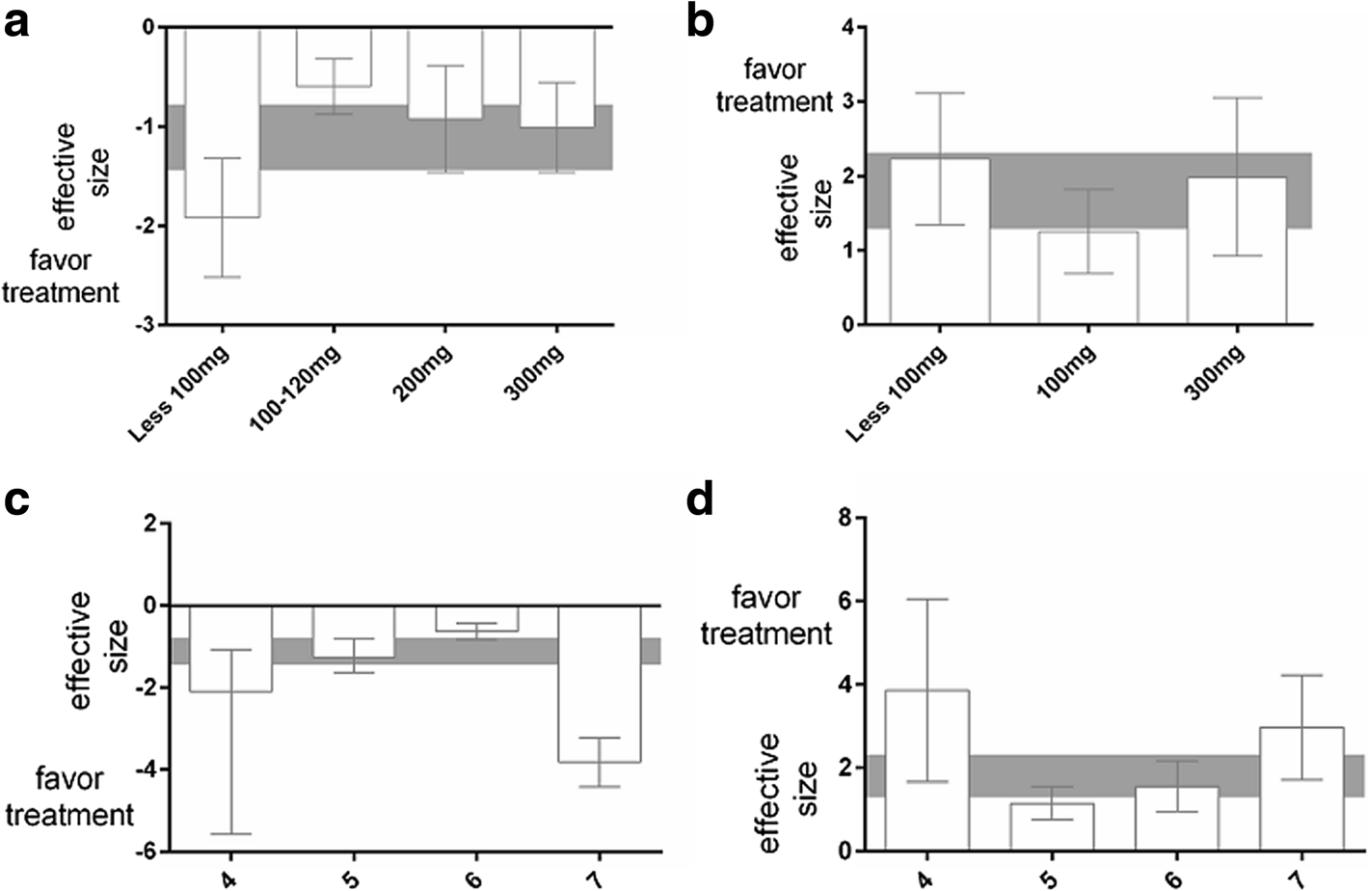
Effect size stratified by the dose of TSG for (**a**) acquisition memory and (**b**) retention memory according to animal gender. Effect size stratified by quality score for (**c**) acquisition memory and (**d**) retention memory. Rey bands represent the 95 % CIs for the global estimated effect sizes

Next, we analysed the efficacy of different doses of TSG on cognitive performance. For both acquisition and retention memory, significant beneficial effects were found for all doses of TSG, with a maximum effect at the lowest dose for both acquisition memory (−1.92, 95 % CI: −2.52 to −1.32) and retention memory (2.23, 95 % CI: 1.34 to 3.11). However, no significant differences among doses were detected for either acquisition memory (*x*^2^ = 8.48, df = 3, *P* = 0.04; Fig. 4a) or retention memory (*x*^2^ = 3.86, df = 2, *P* = 0.15; Fig. 4b).

The effect sizes for acquisition and retention memory were also examined relative to the study quality score. For acquisition memory, the effect size was significantly higher in studies with a quality score of 7 (−3.82, 95 % CI: −4.41 to −3.23) than in those with a quality score of 4, 5, or 6 (*x*^2^ = 101.37, df = 3, *P* < 0.00001); Fig. 4c). No significant differences in effect size were observed relative to study quality for retention memory (*x*^2^ = 12.51, df = 3, *P* = 0.006; Fig. 4d); however, the effect size was highest for studies with a quality score of 4 (3.86, 95 % CI: 1.66 to 6.05).

### Meta-regression analyses

A multivariate random-effects regression with species, sex, model, TSG treatment dose, and study quality score was conducted to further explore the heterogeneity among studies regarding acquisition and retention memory. For acquisition memory, the study quality score was a significant source of heterogeneity (P < 0.05). For retention memory, heterogeneity was independent of all tested factors (Table 6). Finally, we analysed the combined data for acquisition and retention memory to determine whether the study quality score was a significant source of heterogeneity. However, the results showed that the study quality score was not a significant source of heterogeneity for the combined data (coef: −.1396835; 95 % CI: −1.896979-1.61761; t: −0.16).

## Discussion

Systematic reviews of animal studies synthesize the available evidence in an unbiased manner to provide evidence for the potential translational value of effective therapeutic interventions in animal models to humans [17], contribute to models of clinically relevant problems, and facilitate decisions regarding the design and conduct of subsequent human clinical trials.

Systematic reviews of animal studies synthesize existing evidence in an unbiased manner to facilitate decisions regarding the design and conduct of subsequent human clinical trials [18]. To the best of our knowledge, this is the first systematic review and meta-analysis to examine the efficacy of TSG in animal models of AD. This systematic review was performed according to the Preferred Reporting Items for Systematic Reviews and Meta-Analyses [43] flow diagram (Fig. 1). Although small study effects and statistical heterogeneity were present among the included studies, we found that TSG may improve cognitive outcomes relevant to AD [44].

Subgroup analyses of stratified characteristics were assessed to examine the variation in the effects of the intervention, which would suggest that the stratifying characteristic is a crucial factor for heterogeneity and may affect the treatment efficacy. Based on current guidelines, which recommend at least 10 studies per characteristic to stratify subgroups [45], we were able to conduct subgroup analyses of potential sex and species differences, which revealed a higher effect of TSG on acquisition and retention memory in Sprague-Dawley rats than in other species, and less acquisition and retention memory loss following TSG supplementation in studies that used mixed sex groups than in those that used only male or female animals. In addition, TSG treatment was similarly neuroprotective for acquisition and retention memory in both transgenic and non-transgenic models. However, we also found that the lowest dose of TSG provided the greatest benefit in terms of acquisition and retention memory, which is not consistent with a previously described dose-linear response curve [46]. This finding suggests that effect size has been overstated in studies that used lower doses of TSG.

The meta-regression analysis revealed that the heterogeneity was not due to the variables included in the model. Sex, species, animal model, and TSG dose did not affect the heterogeneity of either acquisition or retention memory. The study quality score may explain the heterogeneity in acquisition memory, but the heterogeneity could not be explained by the findings for retention memory or the combined data for acquisition and retention memory. These results may be a consequence of the small sample sizes in those studies and the limited number of studies, reducing the reliability of the analysis. Therefore, we could not conclude that the study quality is dependent on the outcome.

There are some limitations to our present meta-analyses. First, our conclusions are limited by the availability of published trials. We did not include unpublished data in our study. Although we attempted to identify all relevant studies from both Western and Eastern countries, all included studies were conducted within China, which may limit generalizations based on our findings. In addition, it has been reported that some Asian countries, including China, publish unusually high proportions of positive results [27, 47]. Of the studies included in this meta-analysis, most did not report negative findings. We conducted an extensive search of unpublished material in an attempt to obtain negative results, but no unpublished negative studies were identified. We cannot exclude the possibility that studies with negative findings remain unpublished because significant positive findings are more likely to be published than non-significant findings. A meta-analysis based on the published literature may overestimate the efficacy of an intervention [48]. Therefore, publication bias may exist in our meta-analysis, although it seems unlikely that the direction or significance of our findings would be modified by unacknowledged trials.

Second, we observed significant heterogeneity among the study results. Although we used accepted techniques for the meta-regression analysis to identify factors associated with variability in the benefits of TSG treatment, the statistical power of these analyses was relatively low given the number of available trials. Unfortunately, for retention memory, the adjusted R^2^ was 29.72 % due to the limited number of studies. In addition, the covariates included in the model could not explain the heterogeneity more than would be expected by chance. Therefore, it was impossible to accurately determine whether the observed heterogeneity was independent of these factors. The presence of heterogeneity highlights the need for caution in interpreting the present findings [49].

Third, no trial exceeded 6 months in duration, which is relatively short given that patients with AD may require treatment with TSG for decades. Long-term treatment may lead to adverse events or persistent or significant disability/incapacity. Furthermore, we focused on only the effect of TSG on cognitive deficits in animal models of AD, largely due to insufficient data regarding the effect of TSG on neuropathological changes (i.e., β-amyloid plaques and neurofibrillary tangles) in AD.

Fourth, we assessed the methodological quality of studies in accordance with previously described standards for the preclinical development of neuroprotective drugs, with minor modifications [18]. Overall, we found that the quality of the included studies was poor. Many of the studies failed to report blinded outcome assessments, which is recommended for open-label trials to reduce bias. Patient, clinician, and/or assessor awareness of the treatment assignment may influence outcome reporting or measurements and introduce bias [50]. Moreover, although it is important to judge the efficacy of a new drug or therapy, no study reported sample size calculations [51], which should be calculated during the planning phase of the study to evaluate the accuracy of a priori estimates and assist in the design of future experiments [52]. Furthermore, lower quality studies showed a trend towards better retention memory outcomes. Therefore, the global estimated effect of TSG on cognition may be overstated in low quality studies. In addition, studies that included female animals failed to describe their hormonal cycle, which may influence behaviour, body physiology, and cognitive and learning-related performance, and should be accounted for in the experimental design (e.g., by increasing power and/or balancing the randomization of animals across groups) [24, 53].

Fifth, an increasing number of reports on adverse effects and hepatotoxicity of PMT products have been reported in patients [54]. TSG, the main water-soluble active component of *PMT*, was considered the major cause of hepatotoxicity [55, 56]. Nonetheless, no study reported any data on the safety and toxicity of TSG perhaps due to the perception that herbal agents are safe because they are natural products and have a long history of use. Along with the medical use and researches of herbal medicines increased, toxicity and safety of those medicinal materials had become the crucial concerns [57]. It is essential to design additional well-designed and detailed experimental studies to evaluate the safety of TSG before human clinical studies and application.

## Conclusions

Despite its limitations, this systematic review and meta-analysis demonstrates that TSG may reduce cognitive deficits in animal models of AD and indicate a potential therapeutic role of TSG in AD therapy. However, additional scientific experimental studies are needed to evaluate the safety of TSG before human clinical studies and application.

## Abbreviations

AD: Alzheimer’s disease
APP: Amyloid precursor protein
CAMARADES: Collaborative Approach to Meta-Analysis and Review of Animal Data from Experimental Studies
CI: Confidence intervals
df: Degrees of freedom
PM: Polygonum multiflorum Thunb
SMD: Standardized mean difference
TSG: 2, 3, 5, 4′-tetrahydroxystilbene-2-O-β-D-glucoside

## References

[1] G. B. D. Mortality and Causes of Death Collaborators (2014). Global, regional, and national age-sex specific all-cause and cause-specific mortality for 240 causes of death, 1990–2013: a systematic analysis for the Global Burden of Disease Study 2013. Lancet.

[2] Marchalant Y, Baranger K, Wenk GL, Khrestchatisky M, Rivera S (2012). Can the benefits of cannabinoid receptor stimulation on neuroinflammation, neurogenesis and memory during normal aging be useful in AD prevention?. J Neuroinflammation.

[3] Prince M, Wimo A, Guerchet M, Ali GC, Wu YT, Prina M (2015). World alzheimer report 2015. the global impact of dementia. an analysis of prevalence, incidence, cost and trends.

[4] Castellani RJ, Rolston RK, Smith MA (2010). Alzheimer disease. Dis Mon.

[5] Nelson PT, Alafuzoff I, Bigio EH, Bouras C, Braak H, Cairns NJ, Castellani RJ, Crain BJ, Davies P, Del Tredici K (2012). Correlation of Alzheimer disease neuropathologic changes with cognitive status: a review of the literature. J Neuropathol Exp Neurol.

[6] Kortvelyessy P, Gukasjan A, Sweeny-Reed C, Heinze HJ, Thurner L, Bittner DM (2015). Progranulin and amyloid-beta levels: Relationship to neuropsychology in frontotemporal and Alzheimer’s disease. J Alzheimers Dis.

[7] Theofilas P, Polichiso L, Wang X, Lima LC, Alho AT, Leite RE, Suemoto CK, Pasqualucci CA, Jacob-Filho W, Heinsen H (2014). A novel approach for integrative studies on neurodegenerative diseases in human brains. J Neurosci Methods.

[8] Wallace TL, Ballard TM, Pouzet B, Riedel WJ, Wettstein JG (2011). Drug targets for cognitive enhancement in neuropsychiatric disorders. Pharmacol Biochem Behav.

[9] Boada M, Ramos-Fernandez E, Guivernau B, Munoz FJ, Costa M, Ortiz AM, Jorquera JI, Nunez L, Torres M, Paez A. Treatment of Alzheimer disease using combination therapy with plasma exchange and haemapheresis with albumin and intravenous immunoglobulin: Rationale and treatment approach of the AMBAR (Alzheimer Management By Albumin Replacement) study. Neurologia. 2014. doi:10.1016/j.nrl.2014.02.003 10.1016/j.nrl.2014.02.003 25023458

[10] Qin R, Li X, Li G, Tao L, Li Y, Sun J, Kang X, Chen J (2011). Protection by tetrahydroxystilbene glucoside against neurotoxicity induced by MPP+: the involvement of PI3K/Akt pathway activation. Toxicol Lett.

[11] Wang R, Tang Y, Feng B, Ye C, Fang L, Zhang L, Li L (2007). Changes in hippocampal synapses and learning-memory abilities in age-increasing rats and effects of tetrahydroxystilbene glucoside in aged rats. Neuroscience.

[12] Zhou L, Hou Y, Yang Q, Du X, Li M, Yuan M, Zhou Z (2012). Tetrahydroxystilbene glucoside improves the learning and memory of amyloid-beta((1)(−)(4)(2))-injected rats and may be connected to synaptic changes in the hippocampus. Can J Physiol Pharmacol.

[13] Wang T, Yang YJ, Wu PF, Wang W, Hu ZL, Long LH, Xie N, Fu H, Wang F, Chen JG (2011). Tetrahydroxystilbene glucoside, a plant-derived cognitive enhancer, promotes hippocampal synaptic plasticity. Eur J Pharmacol.

[14] Luo HB, Yang JS, Shi XQ, Fu XF, Yang QD (2009). Tetrahydroxy stilbene glucoside reduces the cognitive impairment and overexpression of amyloid precursor protein induced by aluminum exposure. Neurosci Bull.

[15] Li X, Li Y, Chen J, Sun J, Li X, Sun X, Kang X (2010). Tetrahydroxystilbene glucoside attenuates MPP + −induced apoptosis in PC12 cells by inhibiting ROS generation and modulating JNK activation. Neurosci Lett.

[16] Zhang L, Xing Y, Ye CF, Ai HX, Wei HF, Li L (2006). Learning-memory deficit with aging in APP transgenic mice of Alzheimer’s disease and intervention by using tetrahydroxystilbene glucoside. Behav Brain Res.

[17] van Luijk J, Leenaars M, Hooijmans C, Wever K, de Vries R, Ritskes-Hoitinga M (2013). Towards evidence-based translational research: the pros and cons of conducting systematic reviews of animal studies. ALTEX.

[18] Peng W, Yang J, Yang B, Wang L, Xiong XG, Liang Q (2014). Impact of statins on cognitive deficits in adult male rodents after traumatic brain injury: a systematic review. BioMed Res Int.

[19] Vorhees CV, Williams MT (2014). Assessing spatial learning and memory in rodents. ILAR J.

[20] Wolf A, Bauer B, Abner EL, Ashkenazy-Frolinger T, Hartz AM (2016). A comprehensive behavioral test battery to assess learning and memory in 129S6/Tg2576 mice. PLoS One.

[21] Vorhees CV, Williams MT (2006). Morris water maze: procedures for assessing spatial and related forms of learning and memory. Nat Protoc.

[22] Macleod MR, O’Collins T, Howells DW, Donnan GA (2004). Pooling of animal experimental data reveals influence of study design and publication bias. Stroke.

[23] Hooijmans CR, Rovers MM, de Vries RB, Leenaars M, Ritskes-Hoitinga M, Langendam MW (2014). SYRCLE’s risk of bias tool for animal studies. BMC Med Res Methodol.

[24] Hooijmans CR, Pasker-de Jong PC, de Vries RB, Ritskes-Hoitinga M (2012). The effects of long-term omega-3 fatty acid supplementation on cognition and Alzheimer’s pathology in animal models of Alzheimer’s disease: a systematic review and meta-analysis. J Alzheimers Dis.

[25] Sheng C, Peng W, Xia ZA, Wang Y, Chen Z, Su N, Wang Z (2015). The impact of ginsenosides on cognitive deficits in experimental animal studies of Alzheimer’s disease: a systematic review. BMC Complement Altern Med.

[26] Zeng X, Zhang Y, Kwong JS, Zhang C, Li S, Sun F, Niu Y, Du L (2015). The methodological quality assessment tools for preclinical and clinical studies, systematic review and meta-analysis, and clinical practice guideline: a systematic review. J Evid Based Med.

[27] Higgins J, Green S. Cochrane handbook for systematic reviews of interventions, version 5.1.0. Cochrane Collaborations. Chichester, UK: John Wiley & Sons, Ltd. 2011.

[28] Vesterinen HM, Currie GL, Carter S, Mee S, Watzlawick R, Egan KJ, Macleod MR, Sena ES (2013). Systematic review and stratified meta-analysis of the efficacy of RhoA and Rho kinase inhibitors in animal models of ischaemic stroke. Syst Rev.

[29] Zhang L, Xing Y, Ye CF, Ai HX, Wei HF, Li L (2006). Learning memory deficit in APP transgenic model of Alzheimer’s disease and intervention by Shen- wu capsule and its effective component. Chin J Behav Med Sci.

[30] Xing Y, Zhang L, Li L (2006). Effects of tetrahydroxy-stilbene-glucoside on learning and memory abilities and cerebral β-amyloid expression in APP transgenic mice. Chin J New Drugs.

[31] Xie WJ, Zhang L, Wei HF, Chu J (2005). Effects of tetrahydroxy-stilbene-glucoside on gene expression in hippocampus in mice with dementia induced by D- galactose. Chin J Pharmacol Toxicol.

[32] Chu J, Ye CF, Li L, Zhang L (2005). Effects of tetrahydroxystilbene glucoside on learning and memory abilities and neurotrophic factor of brain aging model mice induced by D-galactose. China Pharmacy.

[33] Huang ZS, Xie HY, Nong S, Li T, Zhang SQ (2008). Effects of TSG on learning and memory abilities and expressions of APP and Aβ in hippocampus of mice with dementia induced by D- galactose. J Youjiang Med Coll Nationalities.

[34] Huang R, C., Huang ZS, Li Y, Lai S, Huang J. Ameliorative effects of TSG on learning and memory abilities and free radical scavenging in SMP8 mice. Chin J New Clin Med. 2008;2(9):893–896.

[35] Liu L, Lai S, Li Y, Huang ZS (2012). Eeffects of tetrahydroxy-stilbene-glucoside on learning and memory abilities in SMP8 mice. Mod Chin Med.

[36] Chu J, Ye CF, Li L (2004). Effect of tetrahydroxy-stilbene-glucoside on learning and memory and imflammatory reaction. Tradis Chin Drug Res Clin Pharmacol.

[37] Ye CH, Zhang L, Li B, Ai HX (2003). Effects of 2,3,5,4-tetrahydroxystilbene-2-O-β-d-glycoside on learning and memory function and excitability in Alzheimer’s disease rat induced by model cholinergic damage. Chin J Rehabil Theory Pract.

[38] Ye CH, Wei HF, Zhang L, Zhang L, Li L (2005). Ameliorative effect of 2,3,5,4′-tetrahydroxystilbene-2-O-β-D-glucoside on learning and memory disorder induced by scopolamine in mice. Chin J Clin Rehabil.

[39] Luo HB, Yang JS, Shi XQ (2010). Effect of tetrahydroxy stilbene glycoside on the memory and senile plaque in rat model of Alzheimer disease. Prog Mod Biomed.

[40] Zhao L, Li YL, Zhang L, Li L (2004). Effect of extract of polygonum multiflorum on learning and memory and blood lipids in rats with hypercholesterolemia. Chin Tradit Herb Drugs.

[41] Luo HB, Shi XQ, Yang JS, Fu XF, Yang QD (2012). Expression of autophagy-associated protein induced by p-amyloid and effect of tetrahydroxy stilbene glucoside on it in hippocampus. Chin J Neurol.

[42] Hou Y, Yang Q, Zhou L, Du X, Li M, Yuan M, Zhou Z, Li Z (2011). Tetrahydroxystilbene glucoside improves learning and (or) memory ability of aged rats and may be connected to the APP pathway. Can J Physiol Pharmacol.

[43] Stovold E, Beecher D, Foxlee R, Noel-Storr A (2014). Study flow diagrams in Cochrane systematic review updates: an adapted PRISMA flow diagram. Syst Rev.

[44] Thompson PA, Wright DE, Counsell CE, Zajicek J (2012). Statistical analysis, trial design and duration in Alzheimer’s disease clinical trials: a review. Int Psychogeriatr.

[45] Amos T, Stein DJ, Ipser JC (2014). Pharmacological interventions for preventing post-traumatic stress disorder (PTSD). Cochrane Database Syst Rev.

[46] Dubois B, Zaim M, Touchon J, Vellas B, Robert P, Murphy MF, Pujadas-Navines F, Rainer M, Soininen H, Riordan HJ (2012). Effect of six months of treatment with V0191 in patients with suspected prodromal Alzheimer’s disease. J Alzheimers Dis.

[47] Mimouni M, Krauthammer M, Gershoni A, Mimouni F, Nesher R (2014). Positive results bias and impact factor in ophthalmology. Curr Eye Res.

[48] Schmucker C, Bluemle A, Briel M, Portalupi S, Lang B, Motschall E, Schwarzer G, Bassler D, Mueller KF, von Elm E (2013). A protocol for a systematic review on the impact of unpublished studies and studies published in the gray literature in meta-analyses. Syst Rev.

[49] Gagnier JJ, Morgenstern H, Altman DG, Berlin J, Chang S, McCulloch P, Sun X, Moher D, Ann Arbor Clinical Heterogeneity Consensus Group (2013). Consensus-based recommendations for investigating clinical heterogeneity in systematic reviews. BMC Med Res Methodol.

[50] Kahan BC, Cro S, Dore CJ, Bratton DJ, Rehal S, Maskell NA, Rahman N, Jairath V (2014). Reducing bias in open-label trials where blinded outcome assessment is not feasible: strategies from two randomised trials. Trials.

[51] Gaye A, Burton TW, Burton PR (2015). ESPRESSO: taking into account assessment errors on outcome and exposures in power analysis for association studies. Bioinformatics.

[52] Rutterford C, Taljaard M, Dixon S, Copas A, Eldridge S (2014). Reporting and methodological quality of sample size calculations in cluster randomized trials could be improved: a review. J Clin Epidemiol.

[53] Robertson DA, Savva GM, Kenny RA (2013). Frailty and cognitive impairment--a review of the evidence and causal mechanisms. Ageing Res Rev.

[54] Lei X, Chen J, Ren J, Li Y, Zhai J, Mu W, Zhang L, Zheng W, Tian G, Shang H (2015). Liver damage associated with Polygonum multiflorum Thunb.: a systematic review of case reports and case series. Evid Based Complement Alternat Med.

[55] Yu J, Xie J, Mao XJ, Wang MJ, Li N, Wang J, Zhaori GT, Zhao RH (2011). Hepatoxicity of major constituents and extractions of Radix Polygoni Multiflori and Radix Polygoni Multiflori Praeparata. J Ethnopharmacol.

[56] Bounda GA, Feng YU (2015). Review of clinical studies of Polygonum multiflorum Thunb. and its isolated bioactive compounds. Pharmacognosy Res.

[57] Kale OE, Awodele O (2016). Safety evaluation of Bon-sante cleanser(R) polyherbal in male Wistar rats. BMC Complement Altern Med.

